# Carcinoma-risk variant of EBNA1 deregulates Epstein-Barr Virus episomal latency

**DOI:** 10.18632/oncotarget.14540

**Published:** 2017-01-06

**Authors:** Jayaraju Dheekollu, Kimberly Malecka, Andreas Wiedmer, Henri-Jacques Delecluse, Alan K.S. Chiang, Dario C. Altieri, Troy E. Messick, Paul M Lieberman

**Affiliations:** ^1^ The Wistar Institute, Philadelphia, PA USA; ^2^ Deutsches Krebsforschungszentrum, Heidelberg, Germany; ^3^ Department of Pediatrics and Adolescent Medicine, The University of Hong Kong, Hong Kong

**Keywords:** epstein-barr virus (EBV), EBNA1, nasopharyngeal carcinoma (NPC), survivin, latency

## Abstract

Epstein-Barr Virus (EBV) latent infection is a causative co-factor for endemic Nasopharyngeal Carcinoma (NPC). NPC-associated variants have been identified in EBV-encoded nuclear antigen EBNA1. Here, we solve the X-ray crystal structure of an NPC-derived EBNA1 DNA binding domain (DBD) and show that variant amino acids are found on the surface away from the DNA binding interface. We show that NPC-derived EBNA1 is compromised for DNA replication and episome maintenance functions. Recombinant virus containing the NPC EBNA1 DBD are impaired in their ability to immortalize primary B-lymphocytes and suppress lytic transcription during early stages of B-cell infection. We identify Survivin as a host protein deficiently bound by the NPC variant of EBNA1 and show that Survivin depletion compromises EBV episome maintenance in multiple cell types. We propose that endemic variants of EBNA1 play a significant role in EBV-driven carcinogenesis by altering key regulatory interactions that destabilize latent infection.

## INTRODUCTION

Epstein-Barr Virus (EBV) is a ubiquitous human herpesvirus that infects over 90% of the adult population worldwide [1, 2]. While most EBV infections are benign, the virus is a significant risk factor for a diverse spectrum of human cancers, including endemic forms of Burkitt's lymphoma (BL) and nasopharyngeal carcinoma (NPC). In most adults, EBV establishes a stable latent infection in long-lived memory B-lymphocytes with periodic episodes of lytic reactivation and viral transmission in the oropharyngeal epithelium [3, 4]. In latently infected cells, EBV persists as a stable, non-integrated circular episome that expresses a limited number of viral genes that maintain the latent viral genome and drive host-cell proliferation and survival. However, variations in viral gene expression and genome copy number can be found in different cell and tumor types, suggesting that variable latency control contributes to viral pathogenesis and cancer [5].

EBV-associated cancers are known to occur at elevated rates in geographically restricted regions [2]. These endemic forms of EBV-associated cancers are thought to arise due to a combination of genetic and environmental co-factors in addition to EBV infection [6–8]. The contribution of viral genetic variations has also been considered a potential risk factor, but definitive epidemiological and mechanistic evidence has been lacking. EBV is generally considered to be a single viral species, but several subtypes and genetic polymorphisms have been identified [9, 10]. At least two viral subtypes have been described based on the amino acid variations in the EBNA2 protein [11]. More recent next generation sequencing has revealed a diverse spectrum of genetic variations, including many polymorphisms in EBV coding genes expressed during latent infection and in human tumors [12]. Previous studies had also reported a pattern of polymorphisms in the latency-associated nuclear antigen EBNA1 that may correlate with cancer risk [13, 14].

EBNA1 is the viral encoded DNA binding protein essential for the stable maintenance of the EBV circular genome (referred to as episome) during latent infection [15, 16]. EBNA1 is consistently expressed in all EBV-associated tumors, and is thought to provide a survival function in addition to the maintenance of the viral genome [17, 18]. EBNA1 binds with high affinity to viral origin of plasmid replication (OriP) to drive latent cycle DNA replication and genome segregation required to maintain stable episome copy number in proliferating cells. EBNA1 interacts with numerous host proteins important for DNA replication, episome maintenance, and host survival [19]. Previous studies have identified interactions of EBNA1 with host proteins including EBP2 [20], USP7 [21], casein kinase 2 [22], Tankyrase 1 [23], and others [19], that mediate the different functions of EBNA1 in various cell and latency types. Although EBNA1 is suspected of playing a major role in EBV-associated cancers, its precise function and risk contribution in each type of cancer, beyond maintaining the viral episome, remains poorly defined. Whether variants of EBNA1 contribute to EBV-associated disease is also not known.

Subtypes of EBNA1 enriched in NPC tumors have been identified [24–26]. These EBNA1 subtypes may involve up to 15 amino acid polymorphisms and have been designated as prototypical (P) or variant (V) based on their similarity to B95-8 strain and amino acid 487, with designations P-ala, P-thr, V-pro, and V-leu [9]. The prototypical EBNA1 from the lymphotropic strain B95-8 is termed P-ala, while the majority of NPC-derived EBNA1 is the V-val subtype [25, 26]. Although different subtypes are found in peripheral blood, the V-val subtype is most frequently identified in NPC biopsies [24, 25, 27–30]. Previous biochemical studies found that V-val EBNA1 had no observable differences in DNA binding properties [27] , while others found it had enhanced transcription activation properties in epithelial cell lines [31, 32] and enhanced protection to serum starvation [33]. No previous study has examined the effect of EBNA1 polymorphisms in the context of primary viral infection. Recently, an NPC-derived clonal isolate of EBV, termed M81, has been shown to have distinct properties from prototypical B95-8 strain including an increase in spontaneous lytic reactivation and tropism for epithelial cells [34]. However, the specific contribution of EBNA1 to this phenotype was not addressed. Here, we investigate the biochemical and functional genetic properties of an NPC-derived EBNA1 with V-val subtype. We show that V-val EBNA1 fails to support episome maintenance and suppress lytic cycle gene expression. We propose that these biochemical and functional changes provide a mechanistic explanation for the cancer-risk associated with genetic variants of EBNA1.

## RESULTS

### Structural and biochemical characterization of an NPC-derived EBNA1

**Figure 1:**
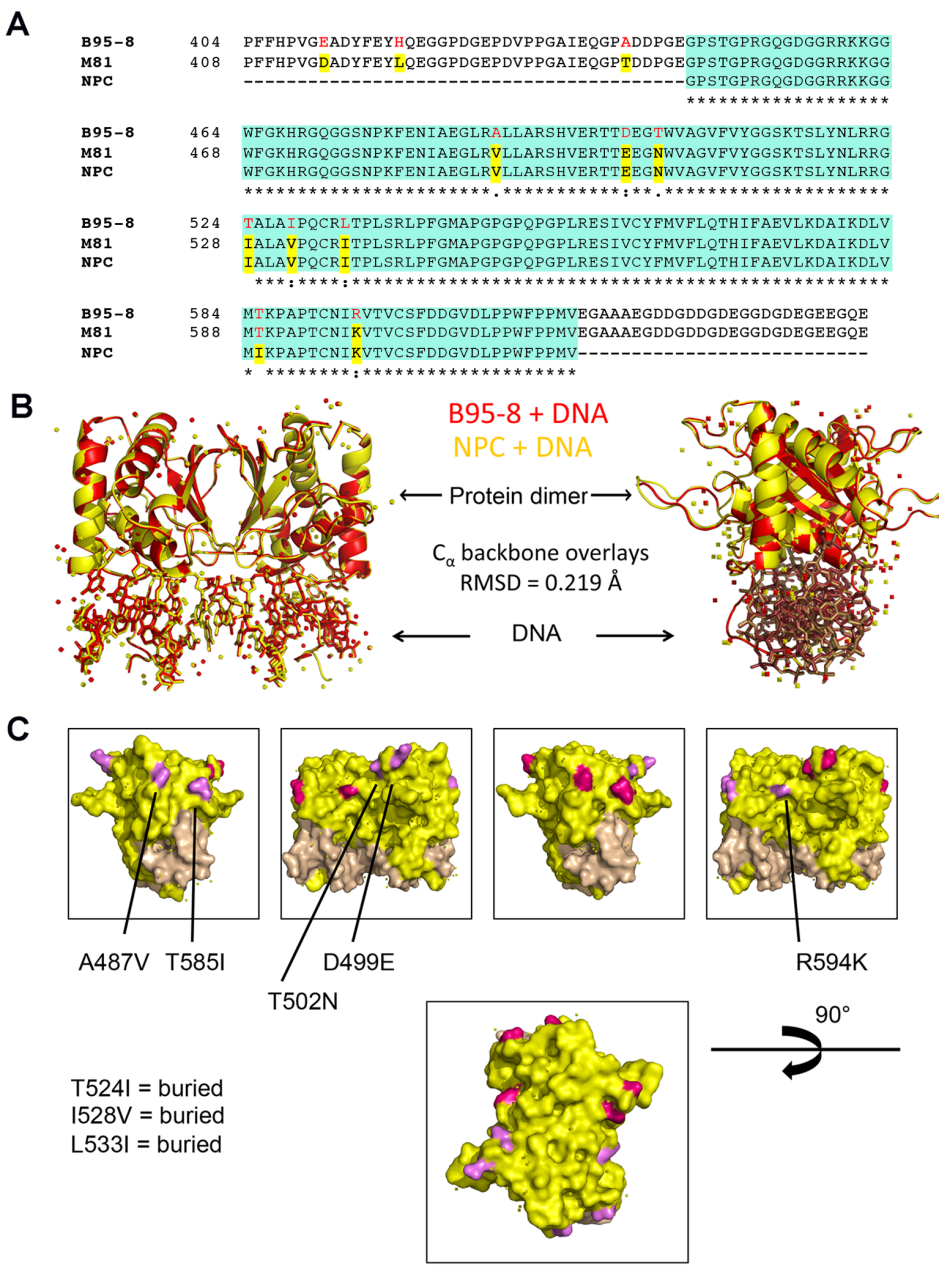
X-ray crystal structures of npcEBNA1 **A**. Alignment of primary amino acid sequence of EBNA1 from B95-8, M81, and NPC derivative. The DNA binding domain included in X-ray crystal structure is highlighted in green, polymorphisms are highlighted in red and yellow. **B**. X-ray crystal structure ribbon projection of NPC EBNA1 (yellow) superimposed on B95-8 EBNA1 (red) both bound to DNA, and with 90° rotation (right). **C**. Polymorphic amino acids that are surface exposed (A487V, T585I, T502N, D499E, R594K) are highlighted in magenta on one EBNA1 monomer and pink in the other EBNA1 monomer. Buried amino acids (T524I, I528V, L533I) are not seen on surface rendering.

To investigate the potential role of amino-acid polymorphisms in the EBNA1 DNA binding domain that may confer NPC risk, we isolated viral DNA from primary EBV positive NPC in patients from Hong Kong [35, 36]. The EBNA1 DBD was amplified by high-fidelity PCR from the HKNPC6-7 variant 2, subcloned, and sequenced. Several polymorphisms were identified that conform to the high-risk forms found in other NPC derived viral strains, including M81 and C666-1 (Figure 1A). These include 10 amino acid substitutions most of which were considered conservative, but several potential variations that could alter function, including T585I and T502N. These polymorphisms are nearly identical to those found in the V-val EBNA1 subtype [31]. The NPC-derived EBNA1 DBD was expressed in E. coli and purified to near homogeneity. We then solved the X-ray crystal structures of NPC and B95-8 EBNA1 DBDs, as well as their co-crystal structures in complex with a cognate DNA binding site derived from EBV OriP (Figure 1B). The structures reveal an overall near-identical fold (RMDS of .219 Å) between NPC and B95-8 EBNA1 DBDs. None of the polymorphic amino acids make direct contact with DNA or altered the DNA binding interface. Amino acids A487V, T585I, T502N, D499E, and R594K are on the exposed EBNA1 surface opposite the DNA binding interface, suggesting that they may alter potential interactions of EBNA1 with other proteins. Amino acids T524I, I528V, and L533I are buried within the protein surface and have no apparent effect on overall structure.

### NPC-derived EBNA1 binds DNA similar to B95-8 EBNA1, but has reduced capacity to stimulate DNA replication

**Figure 2:**
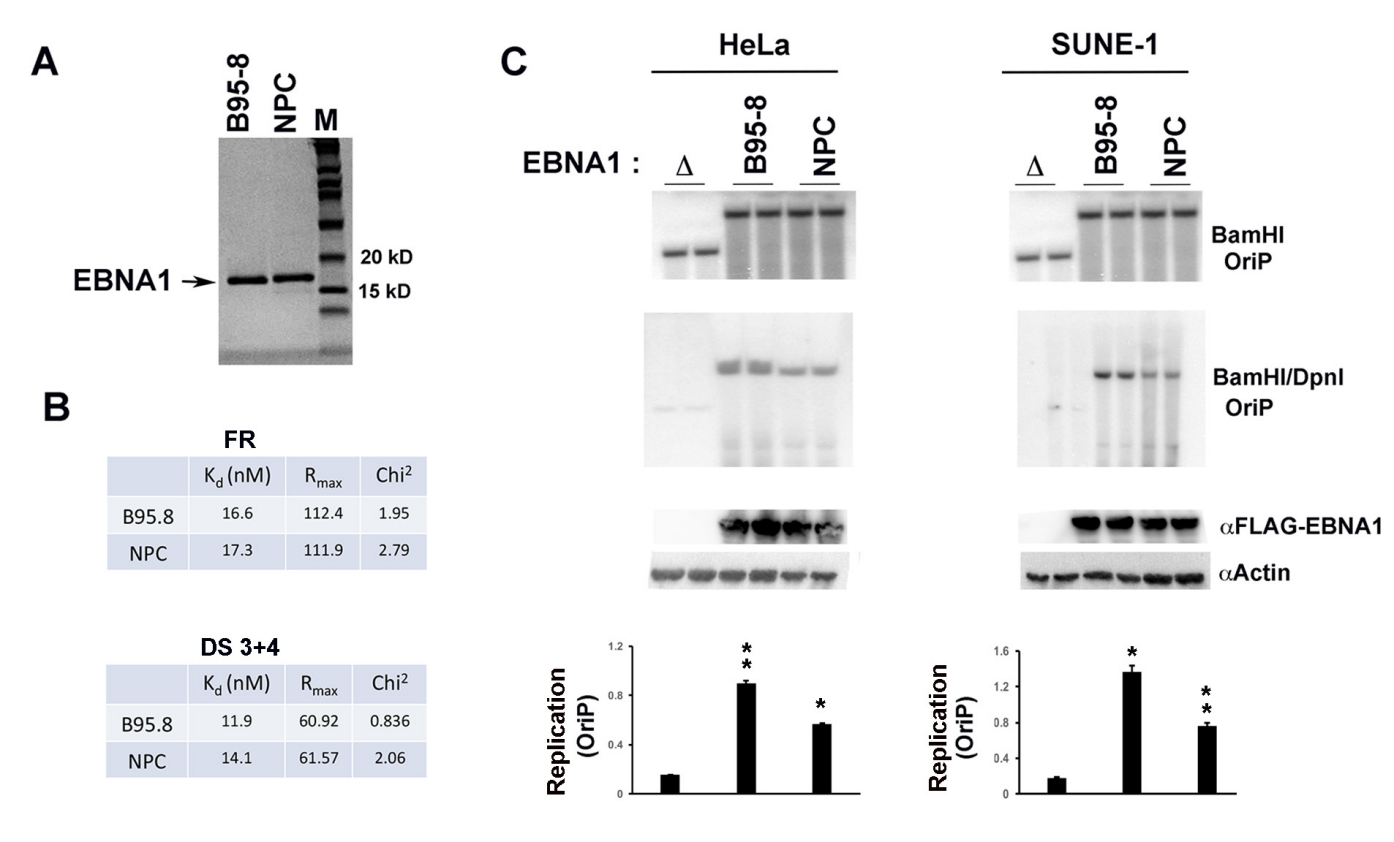
DNA binding and replication properties of npcEBNA1 **A**. SDS-PAGE analysis of 1 µg of purified EBNA1 DBD from B95-8 or NPC variant used for crystallography and *in vitro* DNA binding studies. **B**. Biacore SPR analysis of B95-8 or NPC-derived EBNA1 DNA binding domain interaction with a consensus DNA recognition site found in the family of repeats (FR). The K_d_ (nm), R_max,_ and Chi^2^ for B95-8 and NPC variant of EBNA1 are shown. **C**. OriP-dependent plasmid DNA replication assays were measured with plasmids containing OriP and expressing either full-length (lacking GA-repeats) B95-8 EBNA1 (B95-8), a (NPC), or a deletion of the entire EBNA1 gene (Δ) in either HeLa (left) or SUNE-1 (right) cell lines. DNA replication efficiency was calculated as the ratio of DpnI/BamHI resistant DNA to total BamHI digested DNA. Western blot of FLAG-EBNA1 and Actin controls are shown below. Quantification of at least three biological replicates is shown. p-values were determined by student *t*-test (* < .05, ** < .01). No statistical (ns) difference (*p*-value > 0.05) was observed for B95-8 or NPC EBNA1 in these DNA replication assays.

The NPC- and B95-8 EBNA1 DBDs were next assayed for DNA binding using BIACORE surface plasmon resonance (SPR) assay with two different DNA probes derived from either a single FR site or the paired DS 3+4 binding sites (Figure 2B and Supplementary Figure 1). Both proteins bound with high affinity (16-17 nM for FR, and 11-14 nM for DS 3+4), although NPC-EBNA1 had a slightly reduced affinity for DS 3+4 relative to B95-8 EBNA1 (14.1 *vs* 11.9 nM). We next compared NPC- and B95-8 EBNA1 for their ability to stimulate OriP-dependent DNA replication after transient transfection in two different cell backgrounds (Figure 2C). The DBD from NPC was substituted for the B95-8 DBD in a mammalian expression plasmid containing otherwise full-length B95-8 EBNA1 lacking the Gly-Ala repeats. The same plasmid contained OriP that could be tested for DNA replication using DpnI resistance assay in two different cell types. SUNE-1 is an EBV-negative NPC cell line, while HeLa is an EBV negative cervical carcinoma cell line. We found that NPC EBNA1 stimulated DNA replication but to levels ~60% less than that observed for B95-8 EBNA1 in either HeLa or SUNE-1 cells. This suggests that NPC and B95-8 EBNA1 can bind DNA similarly, but that NPC is compromised for its ability to stimulate OriP-dependent DNA replication.

### NPC-derived EBNA1 is compromised for episome maintenance

**Figure 3:**
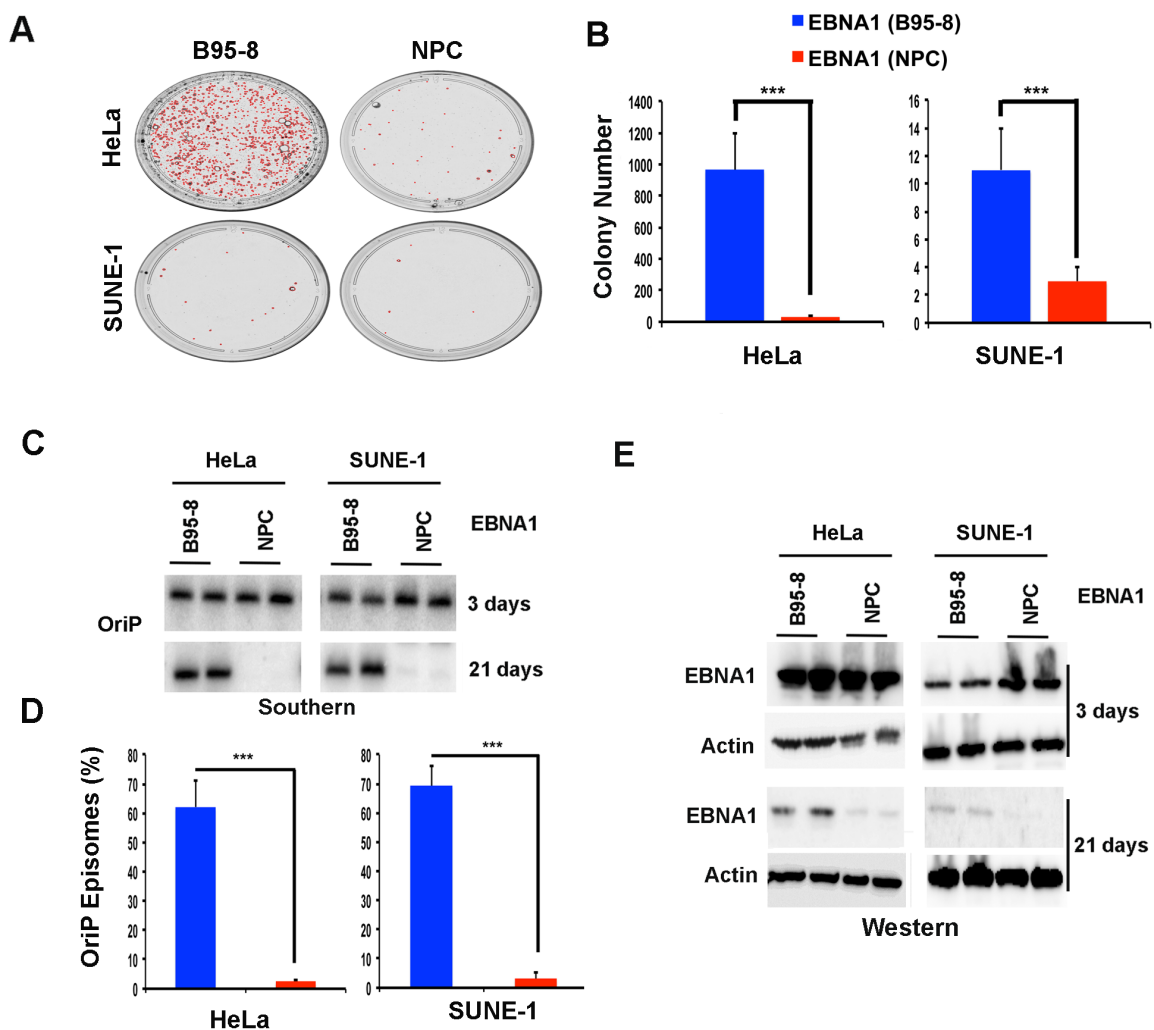
Defective episome maintenance of npcEBNA1 **A**. OriP-containing plasmids expressing hygromycin resistance gene and either B95-8 or NPC-derived EBNA1 DBD were assayed for hygromycin-resistant colony formation in HeLa (left) or SUNE-1 (right) cell lines. Colony number were counted using Image J analysis software for three independent experiments. ** indicates *p*-values < .01 using student *t*-test. **B**. Representative image of colonies counted for panel A. **C**. Episome maintenance assay for OriP-containing plasmids by Southern blot analyses of Hirt lysates. Cells were harvested at 3 and 21 days after transfection in HeLa (left) or SUNE-1 cell lines (right). **D**. Quantification of plasmid maintenance assay for three independent biological replicates. E. Western blot of EBNA1 and Actin in HeLa or SUNE-1 cells after 3 or 21 days post hygromycin selection.

NPC- or B95-8 EBNA1 DBDs were next compared for their ability to support long-term episome maintenance (Figure 3). EBNA1 proteins were expressed from plasmid containing OriP, GFP, and hygromycin resistance. Therefore, we first assayed the relative efficiency of hygromycin-resistant colony formation in HeLa and SUNE-1 cells (Figure 3A and 3B). We found that NPC EBNA1 produced much fewer colonies than B95-8 EBNA1 in HeLa (~20 fold reduction). Both plasmids had reduced colony formation in SUNE-1 cells compared to HeLa, but similar to HeLa, the NPC EBNA1 produced ~4 fold fewer colonies compared to B95-8 EBNA1. We next measured more directly the episome maintenance by Southern blot analysis (Figure 3C and 3D). HeLa or SUNE-1 cells were transfected with NPC- or B95-8 EBNA1-expressing plasmids, selected for hygromycin resistance for 7 days, and then passaged without selection for an additional 21 days (Figure 3C). Consistent with the colony formation assay, we observed a complete loss of OriP-containing episomal DNA by 21 days post-selection in cells expressing NPC EBNA1 relative B95-8 EBNA1 (Figure 3D). Western blot analysis indicated that NPC- and B95-8 EBNA1 proteins were expressed at similar levels at 3 days post-transfection, but loss of EBNA1 correlated with loss of episomes at 21 days post-transfection (Figure 3E). These findings indicate that NPC EBNA1 is severely compromised for episome maintenance relative to B95-8 EBNA1 in two different cell types.

### Generation of a recombinant B95-8 EBV with NPC-derived EBNA1 DNA binding domain (B95-8/npcEBNA1)

**Figure 4:**
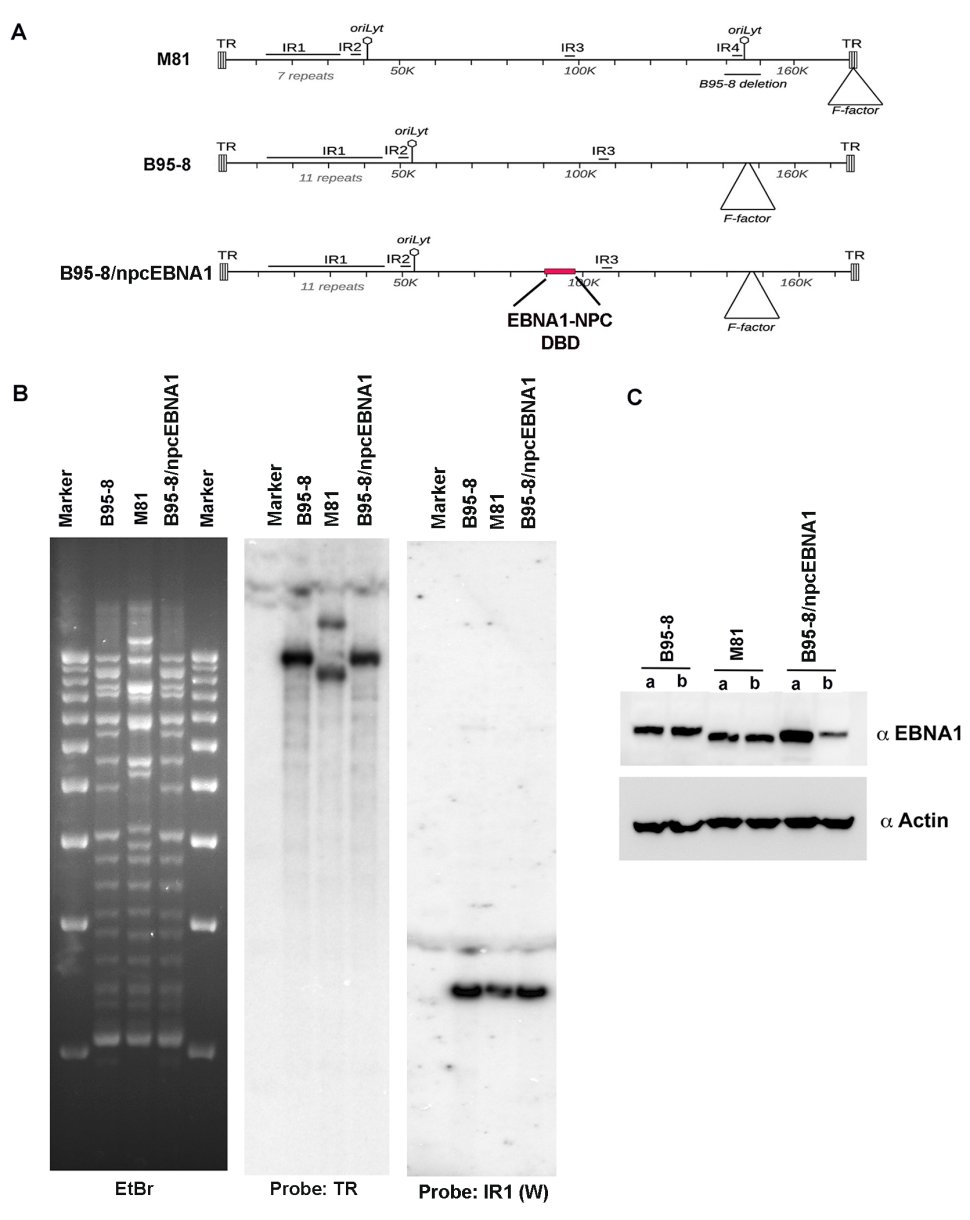
Generation of recombinant B95-8/npcEBNA1 **A**. Schematic map of bacmids containing EBV genomes for M81, B95-8, or B95-8/npcEBNA1. The EBNA1 DBD (aa 454-608) from NPC genome are highlighted in red. **B**. BamHI restriction digest of bacmid genomes for B95-8, M81, or B95-8/npcEBNA1. Ethidium bromide stain agarose gel with 1kb ladder markers (left) was subject to Southern blot hybridization with probe for EBV terminal repeat (TR) (middle panel) or IR1/W repeats (right panel). **C**. Western blot of two independent (a or b) bacmid-transduced 293T producer cell lines for expression of EBNA1 and Actin.

To better assess the effects of the NPC-associated polymorphisms in the EBNA1 DBD in the context of the complete EBV genome and during viral infection, we generated a chimaeric recombinant B95-8 EBV containing the sequence from NPC EBNA1 DBD (Figure 4A). A gene-block containing the NPC EBNA1 was generated and introduced by two-step red recombination methods into the B95-8 bacmid. The correct recombinant (designated B95-8/npcEBNA1) was validated and shown to have indistinguishable restriction digestion pattern from parental B95-8 bacmid, as well as intact terminal repeats (TR), and internal repeat 1 (W repeats) as measured by Southern blot (Figure 4B). We also confirmed that NPC EBNA1 DBD was correct by direct Sanger sequencing of the EBNA1 gene. The B95-8 bacmid was compared to the NPC-derived M81 bacmid, which contains nearly identical substitutions in EBNA1 DBD as NPC EBNA1. These bacmids were introduced into 293T cells and shown to express EBNA1 proteins of the expected molecular mass (Figure 4C). These 293T cell lines were then used to generate infectious EBV for subsequent study of primary B-cell infection and transformation.

### Inefficient immortalization of primary B-lymphocytes by viral genomes with NPC EBNA1 DBD

**Figure 5:**
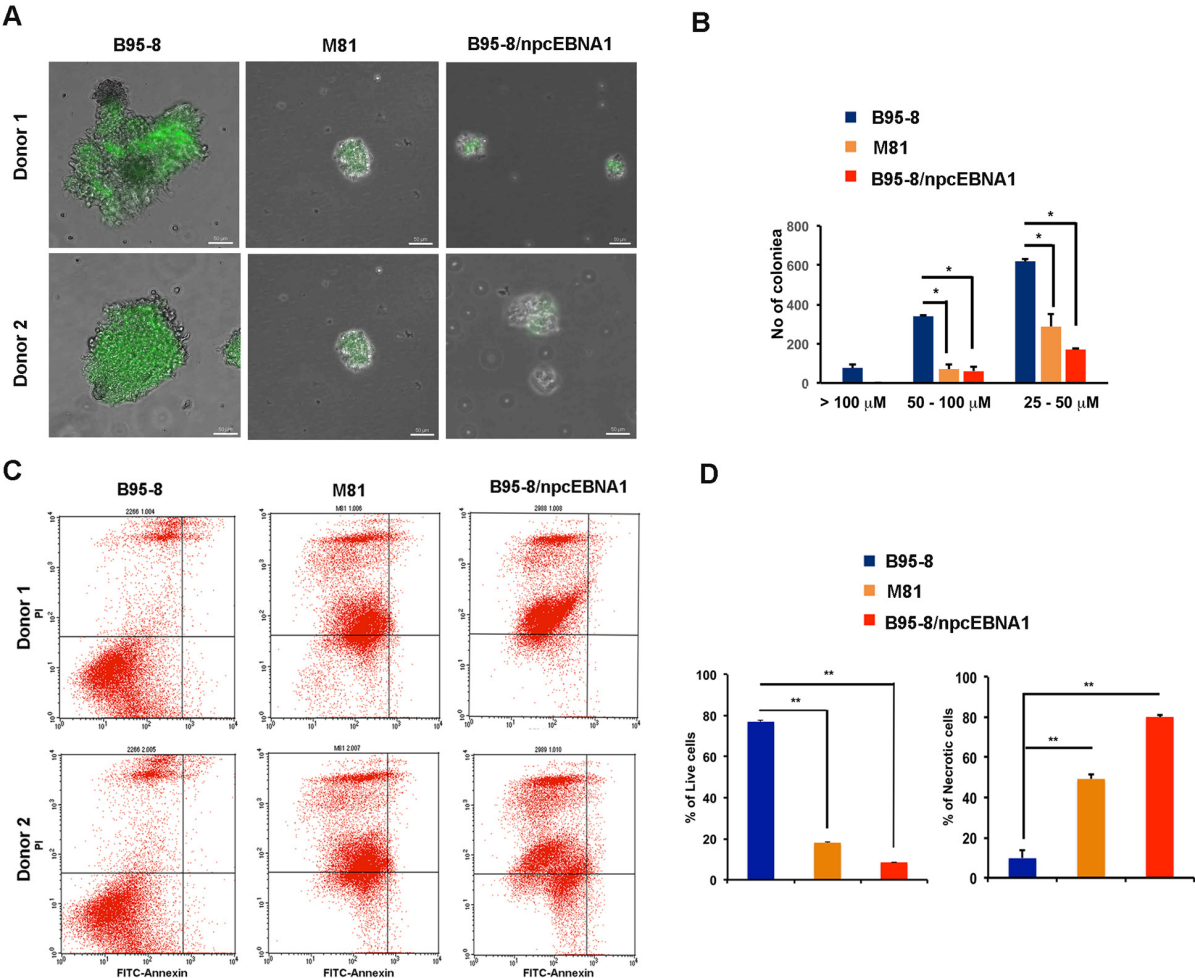
Defective B-cell blast formation by B95-8/npcEBNA1 Bacmid-derived virus for B95-8, M81, or B95-8/npcEBNA1 were assayed at 2 weeks post-infection of primary B-lymphocytes. **A**. GFP positive B-cell blasts for two independent donors were analyzed by high-throughput microscopy. **B**. The number of colonies imaged by microscopy with diameters of > 100, 50-100, or 25-100µM were quantified by Image J (panel B). **C**. B-cell blasts at 4 weeks post-infection were analyzed by FACS for apoptosis using propidium idodide (PI) (x-axis) and annexin V (y-axis). **D**. The percentage of live and necrotic cells assayed by FACS were quantified for two independent donors and three independent biological replicates.

Primary B-cells from two different donors were infected with equal infectious units of recombinant virus from B95-8, M81, or B95-8/npcEBNA1. We observed that B95-8 was highly efficient at producing large and robust B-cell blasts within 2 weeks after infection with two separate donors. In contrast, both M81 and B95-8/npcEBNA1 produced small blasts that were slow to expand until ~4 weeks post-infection (Figure 5A and 5B). FACS analysis of these later B-cell blasts revealed high levels of annexin V positive apoptotic cells in M81 and B95-8/npcEBNA1 virus infection (Figure 5C and 5D). Quantitation of the average size of the B-cell blasts (Figure 5B), as well as the percent of live and necrotic cells (Figure 5D), confirmed that B95-8 transforms primary B-cells more efficiently than does M81 or B95-8/npcEBNA1 virus.

### Defects in suppressing lytic cycle gene expression during early phase of B-cell infection by NPC EBNA1

**Figure 6:**
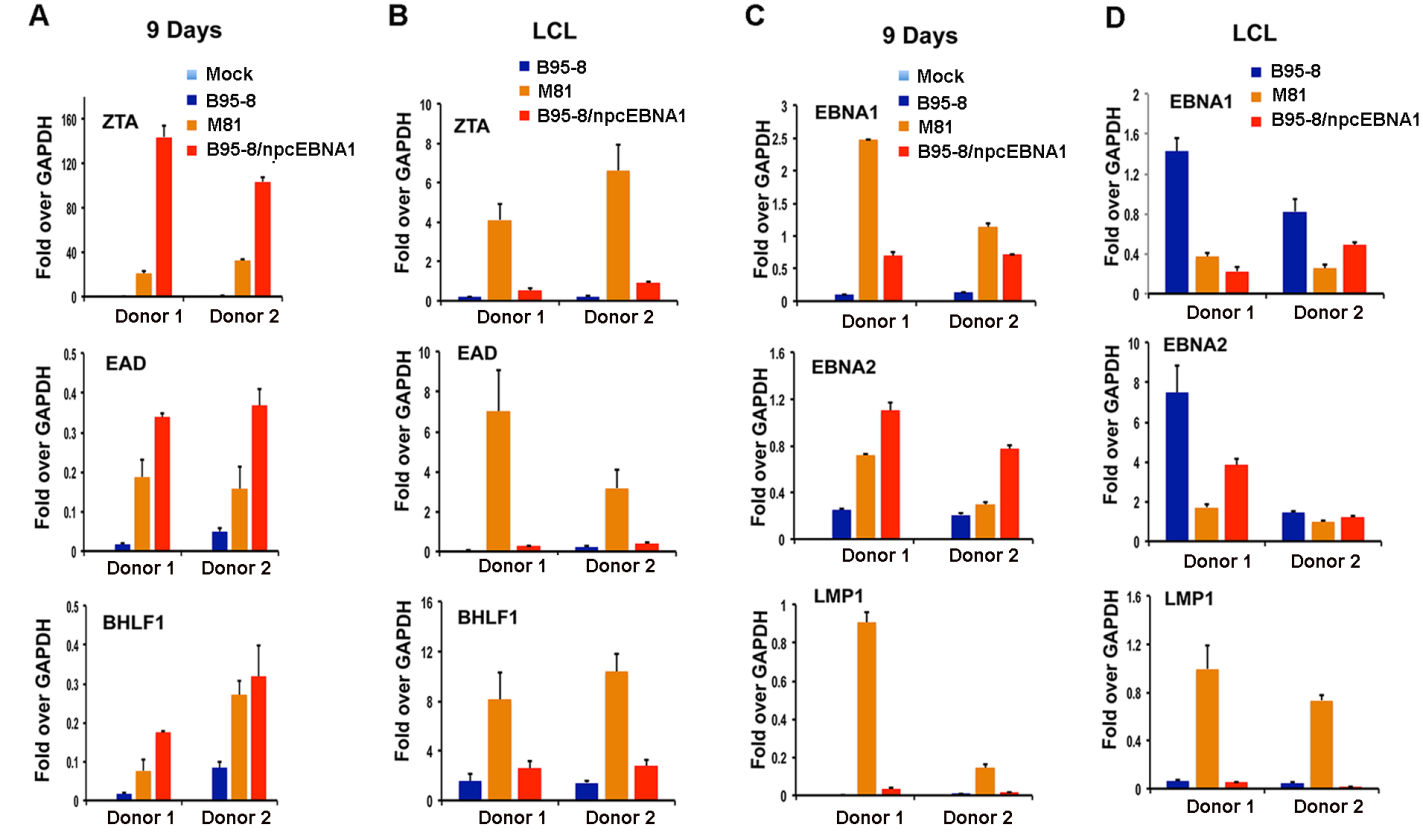
Variant viral transcription during early stages of B-cell infection by B95-8/npcEBNA1 RT-PCR of primary B-cells infected with either mock, or bacmid derived B95-8, M81, or B95-8/npcEBNA1 virus at either 9 days post-infection (panels **A** and **C**) or for established LCLs at 2 months post-infection (panels **B** and **D**). EBV lytic transcripts for ZTA, EA-D, or BHLF1 (panels **A** and **B**), or latency associated transcripts for EBNA1, EBNA2, or LMP1 (panels **C** and **D**) are shown relative to GAPDH. RT-PCR is shown as the average for three replicates for two independent donors. Error bars represent standard deviation from the mean.

Primary B-cells infected with B95-8, M81, or B95-8/npcEBNA1 were assayed by RT-PCR at either 9 days post-infection or when LCLs were established. LCLs were established at later time points (8-12 weeks) for M81 and for B95-8/npcEBNA1 relative to B95-8 (3-4 weeks). We found that lytic transcripts for ZTA, EA-D, and BHLF1 were highly upregulated at 9 days post-infection in B95-8/npcEBNA1 relative to B95-8 for two independent donors. M81 was also elevated for lytic transcripts at this time point, but not to the same extent at B95-8/npcEBNA1 virus (Figure 6A). Interestingly, in established LCLs, the lytic transcription for B95-8/npcEBNA1 virus returned more to that of B95-8 levels, while the M81 LCLs remained elevated (Figure 6B). Latency transcripts were also deregulated, with especially high levels of LMP1 in M81 virus infections at 9 days (Figure 6C) and in established LCLs (Figure 6D) relative to the other two viruses. These results suggest that B95-8 virus carrying EBNA1 DBD fails to suppress lytic transcripts at early stages of B-cell infection. M81 may have additional variations in gene expression that account for the elevated expression of LMP1 in established LCLs.

### Failure to maintain circular episomes by NPC EBNA1

**Figure 7:**
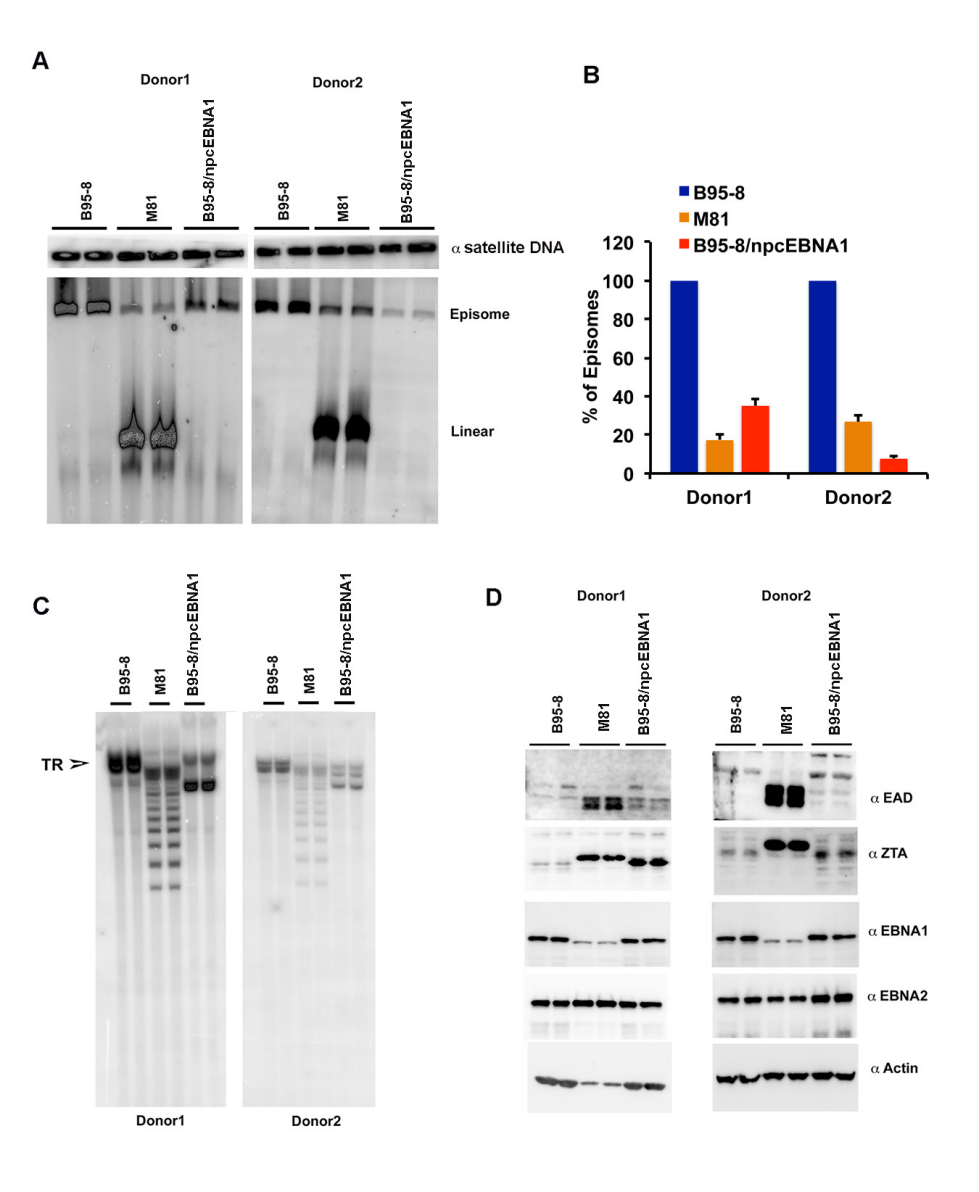
Low episome copy number and terminal repeat instability in LCLs with B95-8/npcEBNA1 **A**. PFGE analysis of LCLs generated with recombinant B95-8, M81, or B95-8/npcEBNA1 virus. Samples are run as technical replicates for two independent donor generated LCLs. Cellular α-satellite DNA is shown as loading control above each lane. **B**. Quantitation of EBV episomes relative to α-satellite DNA for PFGE shown in panel A. **C**. Southern blot analysis of EBV terminal repeats after digestion with BamHI for B95-8, M81, or B95-8/npcEBNA1 generated LCLs. **D**. Western blot for EAD, ZTA, EBNA1, EBNA2, and Actin for B95-8, M81, or B95-8/npcEBNA1 generated LCLs.

To further investigate the molecular basis for the poor transforming efficiency of M81 and B95-8/npc EBNA1, we assayed the early stage LCL (8 weeks) for EBV episome stability and structure (Figure 7A-7C, Supplementary Figure 2). EBV episome stability was first assayed by pulse-field gel electrophoresis (PFGE) and Southern blot analysis. We found that LCLs from B95-8 had exclusively episomal genomes, while LCLs form M81 had a large proportion of linear genomes. The B95-8/npcEBNA1 LCLs were mostly episomal, but had lower copy number than did B95-8 (Figure 7A and 7B). We also measured the structure of the terminal repeats, which can be altered due to background lytic replication, as well as by genomic instability (Figure 7C). We found that B95-8 had a single major band consistent with a clonal form of episomes, while M81 had a degenerate number of TR, indicative of lytic replication. Interestingly, B95-8/npcEBNA1 genomes from LCLs had an intermediate terminal repeat structure, with a doublet showing a major species below the full length B95-8 TR (Figure 7C). This degenerate TR was not detected in the bacmid derived genomes (Figure 4B), indicating that these changes occur during the early stages of latency establishment in lymphoblastoid cultures. Similar results were obtained with a second, independent recombinant B95-8/npcEBNA1 virus (Supplementary Figure 2). This suggests that B95-8/npcEBNA1 virus has a defect in establishing or maintaining a stable latent episome, although not as extensive a defect as found in the M81 strain. Western blot analysis of these LCLs showed that M81, and to a lesser extent B95-8/npcEBNA1, expressed an increase levels of ZTA and EAD relative to B95-8 (Figure 7D), consistent with RT-PCR analysis (Figure 6).

### Identification of Survivin as an EBNA1 interacting protein compromised by NPC EBNA1 polymorphism

**Figure 8:**
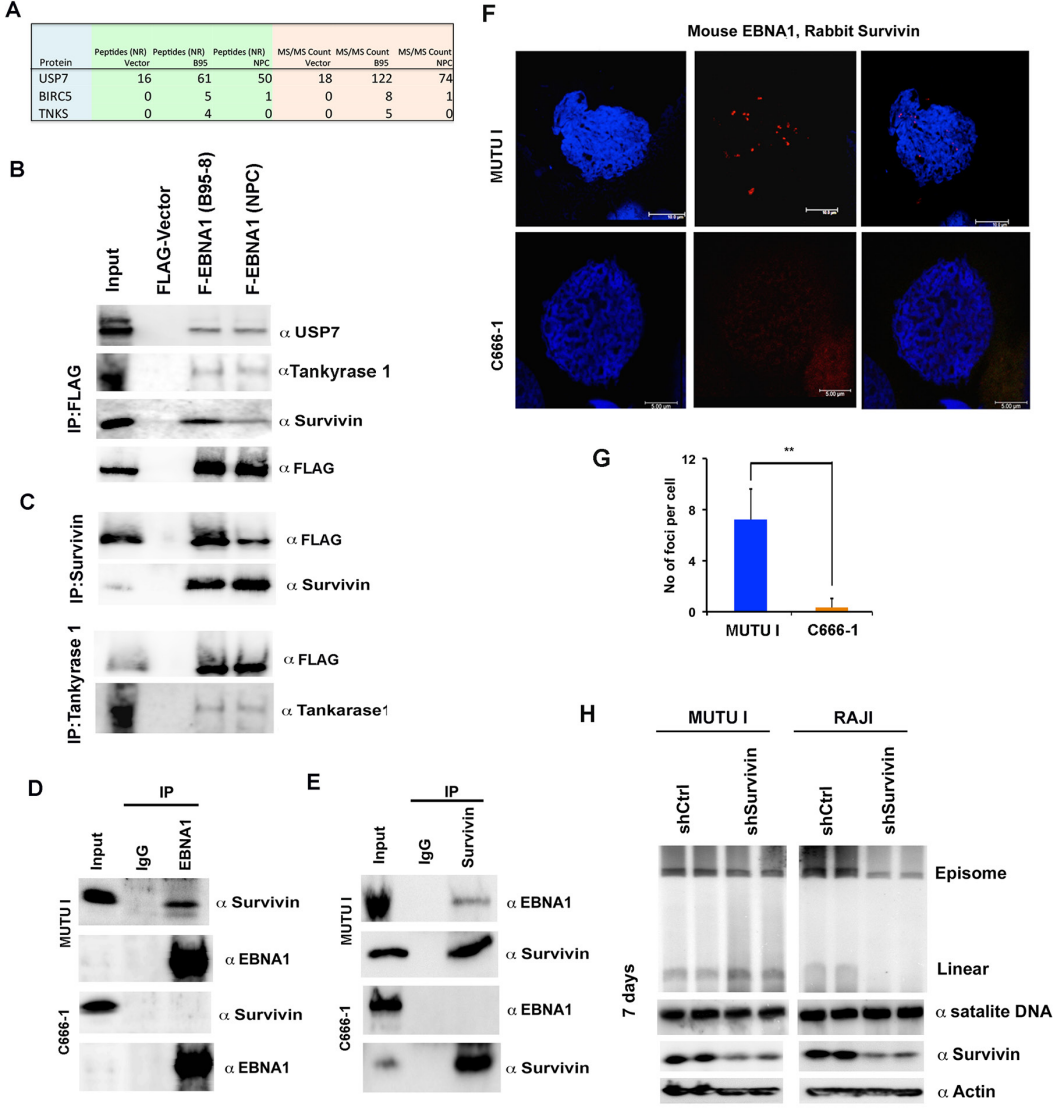
NPC-derived EBNA1 is compromised for interaction with Survivin **A**. LC-MS analysis of proteins associated with FLAG-EBNA1 (F-EBNA1) from B95-8 or with the NPC DNA binding domain. Unique peptides and spectral counts are shown for USP7, BIRC5 (Survivin), and Tankyrase 1 (TNKS). **B**. Western blot analysis of FLAG IP from HeLa cells with stable expression of FLAG-vector, F-EBNA1 (B95-8) or F-EBNA1 (NPC) probed with antibody to USP7, Tankyrase 1, Survivin, or FLAG. **C**. Extracts from stable HeLa cells shown in panel B, were subject to IP with antibody to Survivin probed with antibody to FLAG or Survivin (top panel). Extracts from stable HeLa cells shown in panel B were subject to IP with antibody to Tankyrase 1 probed with antibody to FLAG or Tankyrase1 (lower panel). **D**. MutuI cell (top panels) or C666-1 cell (lower panels) extracts were subject to IP with antibody to EBNA1 or control IgG, and then assayed by Western blot with antibody to Survivin or EBNA1, as indicated. **E**. Same as in panel D, except IP with Suvivin or control IgG. **F**. *In situ* Proximity Ligation Assay (*in situ* PLA) is shown for interphase cells from either MUTU-I or C666-1 using mouse anti-EBNA1 and rabbit anti-Survivin. **G**. Quantification of *in situ* PLA for n > 100. ** *p*-value < .01 using student *t*-test. **H**. PFGE analysis of EBV episomes and linears at 7 days post-transduction with lentivirus shRNA for shControl or shSurvivin in MUTU I or RAJI cell lines. Human α satellite DNA is used for DNA loading control Western blot of Survivin and Actin are shown below for each cell at 7 days post-transduction.

To determine if any EBNA1 interacting proteins may account for the NPC phenotype, we performed a mass spectrometry proteomics analysis (LC/MS/MS) comparing NPC to B95-8 EBNA1. We compared the relative intensity of signal detected by LC/MS/MS for proteins isolated in association with FLAG-EBNA1 with B95-8 or NPC DBD. As expected, we identified several proteins previously known to interact with EBNA1, including USP7 and Tankyrase 1 (Figure 8A). These proteins bound to NPC and B95-8 EBNA1 with similar avidity in LC/MS/MS and this was confirmed by Western blot (Figure 8B and 8C). We identified Survivin (BIRC5) as a candidate interacting protein that had higher LC/MS/MS counts in B95-8 relative to NPC EBNA1. We confirmed by Western blot that Survivin co-immunoprecipitated with B95-8 EBNA1 with higher avidity than did NPC EBNA1 (Figure 8B and 8C). To determine if endogenous EBNA1 interacted with Survivin in EBV infected tumor cells, we performed coIPs in MUTU I and C666-1 cells (Figure 8D and 8E, and Supplementary Figure 3). C666-1 cells have NPC variant V-val EBNA1, while MUTU I is an African Burkitt lacking these same polymorphisms. IP with αEBNA1 pulled down Survivin in MUTU I, but not C666-1, while controls for EBNA1 IP and Survivin inputs were similar in both cell types (Figure 8D). The reverse IP showed the same trend, with αSurvivin IP pulling down EBNA1 in MUTU I, but not C666-1 (Figure 8E). To determine whether EBNA1 colocalizes with Survivin at mitotic chromosome *in vivo*, we performed *in situ* proximity ligation assay (PLA) using PCR to amplify ligated DNA oligos on antibodies to EBNA1 or Survivin (Figure 8F-8G). We compared EBV positive BL cells (MUTU I) with NPC derived cells (C666-1) assaying both mitotic arrested (Figure 8F) and asynchronous (Supplementary Figure 4) cells. We found strong PLA signals for the interaction of EBNA1 and Survivin in MUTU I cells, but not in C666-1. PLA signals were strongly associated with DAPI stained chromosomes in metaphase (Figure 8F), as well as in non-mitotic cells (Supplementary Figure 4). Quantification of multiple PLA images revealed a highly consistent and significant difference between MUTU I and C666-1 cells (Figure 8G). Control IF experiments indicated that EBNA1 and Survivin signals could be detected in both cell types (Supplementary Figure 5). These findings indicate that EBNA1 and Survivin interact in MUTU I BL cells, but that this interaction is not readily detected in C666-1 NPC cells.

To determine if Survivin contributes functionally to the maintenance of EBV genomes in BL cells, we assayed the effect of Survivin shRNA depletion in two different BL cells, MUTU I and Raji, which have very high levels of EBV episomes compared to NPC cell line C666-1 (Figure 8H). We assayed episome maintenance by PFGE at 7 days post-lentivirus transduction, since longer times led to a large number of apoptotic cells (data not shown). Western blot revealed that Survivin depletion was ~50% at 7 days. PFGE revealed a partial loss of EBV episomes in both MUTU I (~50% loss) and Raji (~80% loss) (Figure 8H and Supplementary Figure 6). These data suggest that Survivin can associate with EBNA1, and is important for EBV episome maintenance in BL cells.

## DISCUSSION

Genetic polymorphisms in the EBV genome have been identified, but their contribution to pathogenesis has not been fully assessed [35–37]. The risk factors for endemic NPC include geographical and genetic components [38, 39], as well as high loads of EBV DNA and antibodies to viral lytic proteins [40, 41]. Variants of EBV genomes and gene products have been identified, but their specific contribution to cancer risk has remained obscure. Here, we provide biochemical and genetic evidence that polymorphisms in the EBNA1 DBD contribute to the risk phenotype associated with EBV in endemic NPC.

### Molecular and functional properties of NPC-derived EBNA1

EBNA1 genes derived from NPC tumors typically have 6-8 amino acid substitutions relative to B95-8 strain at non-random positions within the DBD. Here, we have investigated the structural, biochemical, and functional properties of an NPC-derived EBNA1 DBD with a V-val signature. The X-ray crystal structures of free and DNA-bound NPC EBNA1 revealed that the overall fold of NPC and B95-8 EBNA1 DBD are highly similar, with most of the polymorphic amino acids on protein surface away from the DNA-binding interface (Figure 1). We observed only minor difference in the DNA binding properties of NPC or B95-8 EBNA1 DBDs, as may be expected given the mutations fall on the surfaces away for the DNA binding interface (Figure 2A-2B). However, NPC EBNA1 DBD was found to be defective for DNA replication (Figure 2C and 2D) and episome maintenance (Figure 3). We engineered the NPC EBNA1 DBD into a B95-8 genome (Figure 4) and found that it conferred properties similar to the M81 strain, especially during the early stages of primary B-cell infection (Figures 5-6). This included small blast formation, increase apoptotic and necrotic cells (Figure 5), and high-levels of lytic gene expression at 9 days post-infection (Figure 6). Stable LCLs took longer to establish with NPC-derived EBNA1 DBD and had reduced episome copy number and unstable terminal repeat numbers (Figure 7). Finally, we identified Survivin as a host cell protein that interacted preferentially with B95-8 compared to NPC EBNA1 (Figure 8).

### Significance of EBNA1 subtypes in NPC

The V-val variant has been isolated most consistently from NPC biopsies. While V-val is also detected in peripheral blood of endemic populations, its enrichment in NPC tumor cells remains significant since other EBNA1 variants are found in peripheral blood of the same and control patients from the same location [24, 42]. Previous studies have suggested that the V-val variant of EBNA1 provides selective tumor growth advantage [33]. Unexpectedly, we found that the NPC-variant of EBNA1 functions less efficiently in episome maintenance than B95-8 DBD, even in NPC cell line SUNE-1. It is possible that NPC-variants are optimized for replication and maintenance for a co-evolved variant OriP from the same genome, and future experiments will need to examine the OriP genetic variation in conjunction with EBNA1. However, our data is more consistent with the observation that an NPC strain, such as M81, which contains an NPC-derived EBNA1 and OriP, is compromised for suppression of lytic genes and is maintained poorly as a latent episome in B-lymphocytes. This then raises the pathophysiological question as to how a deficiency in B-cell episome maintenance could increases the risk of NPC.

One mechanism through which defective episome maintenance may increase NPC risk is by failing to establish stable epigenetic suppression of lytic gene expression. Previous studies have indicated that episome maintenance is mechanistically linked to the establishment of chromatin structures and epigenetic modifications that stabilize viral chromosome transmission during mitosis and viral gene expression during latency [43]. Failure to establish stable epigenetic repression leads to the aberrant expression of lytic cycle genes and lytic DNA replication. Thus, a failure to establish stable latent epigenetic control of latent episomes could account for many of the observed properties associated with EBV in NPC patients, including increased titer to lytic antigens and aberrant infection in nasopharyngeal epithelium [44, 45].

### Survivin as a novel EBNA1 interacting protein

EBNA1 has been found to interact physically with several host proteins [15]. Here, we have identified Survivin as a new interacting partner for EBNA1. We found that Survivin co-purified and co-precipitated with EBNA1 from cells that maintain a stable EBNA1-OriP episome (Figure 8). Survivin is unlikely to have a high-affinity for EBNA1 in the absence of OriP, as previous studies did not identify Survivin as an EBNA1 interacting partner in cell expressing ectopic EBNA1 lacking OriP [19]. Survivin is a multifunctional protein that regulates chromosome segregation during mitosis [46] and inhibits apoptosis in most cancer cells [47]. Survivin is a member of the IAP family known to inhibit cellular apoptosis pathways [47]. Survivin is upregulated in many cancers, including EBV associated NPC, and during EBV immortalization of B-lymphocytes [48]. At least several EBV proteins have been implicated in the up regulation of Survivin, including EBNA1 [49] and LMP1 [50, 51]. Survivin is also a member of the chromosome passenger complex (CPC) that regulates Aurora B and Polo-like kinase 1 (PLK1) activity at kinetochores during mitosis [46]. Our *in situ* PLA reveals that EBNA1 and Survivin preferentially interact at only a few locations found on metaphase chromosomes. These PLA interaction foci were not readily detected in NPC cell line C666-1 that has a V-val subtype of EBNA1 and lower episome copy number. Our data suggests that EBNA1 and Survivin colocalize in cells where episomes are maintained efficiently, and at numbers consistent with episome copy numbers. Furthermore, depletion of Survivin caused a reduction in episome maintenance in two BL cell lines that typically maintain stable latent episomes. Failure to interact with Survivin correlates with inefficient episome maintenance. Taken together, we propose that Survivin functions with EBNA1 at OriP to establish chromosome passenger like-features necessary for episome maintenance.

### NPC-derived EBNA1 fails to suppress early lytic transcripts during B-cell immortalization

Primary infection of B-lymphocytes with M81 or B95-8/npcEBNA1 virus showed atypical elevation in lytic gene expression at 9 days post-infection. Precisely how NPC EBNA1 DBD deregulates lytic transcription is not known. Previous studies demonstrated that shRNA depletion of EBNA1 leads to an increase in lytic gene expression, suggesting the EBNA1 has a direct or indirect role in transcriptional repression of lytic genes [18]. It is possible that the defect in episome maintenance by NPC EBNA1 is mechanistically linked to the failure to repress lytic transcripts in the context of the complete viral genomes. Instability of terminal repeats (Figure 7C) may also be linked to the defects in episome maintenance, as well as the derepression of lytic transcription. The defective interaction with Survivin (Figure 8) could be the underlying biochemical basis for these phenotypes, as a failure to assemble appropriate mitotic chromatin at OriP may compromise episome maintenance and repression of lytic gene expression.

Viral genetic variation is known to have significant impact on pathogenesis, but the contribution of EBV genetic variation has not yet been mechanistically linked to increase risk of NPC. Our findings implicate polymorphisms in the EBNA1 DBD as potential drivers of EBV lytic gene expression during latency and risk factors for NPC. This genetic variation in EBNA1 DBD confers a phenotype that is consistent with observed properties of EBV in NPC patients and tumors. Our findings provide a mechanistic basis and new paradigm for the potential contribution of EBNA1 polymorphisms to the etiology of endemic NPC.

## EXPERIMENTAL PROCEDURES

### Cells, plasmids, shRNA and antibodies

EBV-positive Burkitt lymphoma cell lines MUTU I (gift of Jeff Sample (Penn State University, Hershey), RAJI (gift of Diane Hayward, Johns Hopkins University) and EBV bacmid immortalized lymphoblastoid cell lines (LCLs) (generated at the Wistar Institute) were grown in RPMI medium (Gibco BRL) containing 15% fetal bovine serum, and antibiotics penicillin and streptomycin (50U/ml). 293T (ATCC), Hela (ATCC) and EBV-negative NPC-derived cell line SUNE-1 (gift of Maria Li Lung, Hong Kong University) were cultured in Dulbecco's modified Eagle's medium (DMEM) with 10% fetal bovine serum and antibiotics. All the cells were cultured at 37 °C and 5% CO2 environment. Mammalian expression vector for Flag-EBNA1 contains B95-8 EBNA1 lacking Gly-Ala repeats under the control of CMV-3XFLAG promoter in a plasmid derived from pREP10 (Clonetech) containing OriP, GFP, and hygromycin resistance. pFLAG-npcEBNA1 is identical to pFLAG-EBNA1, except with the V-val polymorphisms in the DNA binding domain (A487V, D499E, T502N, T524I, I528V, L533I, T585I, R594K). Small hairpin RNAs (shRNAs) for Survivin (shSurvivin),and the control (shControl) were obtained from the Sigma/TRC collection of targeted shRNA plasmid library (TRC no. 73718,73719,73720, 73721, and SCHOO2). Lentivirus particles were generated in 293T-derived packaging cells. Mouse monoclonal anti-Survivin (Novus biologicals; NB500-204), mouse monoclonal anti-actin (Sigma A3854), mouse monoclonal anti-EBNA1 (Acris BM3127) rat anti-EBNA2 (Fisher 50175912), mouse anti-LMP1 (Dako M0897), Rabbit polyclonal anti-EBNA1 and anti-Zta antibody (in house) were used for Western blotting.

### Protein purification of EBNA1 DBD

Residues 459 - 607 of EBNA was cloned into a modified pET DUET vector containing the yUBE3 gene to encode the Hexa-His tag with yeast small ubiquitin-like modifier (SUMO) protein fused to EBNA1 (459-607). BL21(DE3) *E. coli* cells were transformed with plasmid, grown in the presence of 100 µg/mL ampicillin and induced *via* autoinduction. Cell pellets were resuspended in 20 mM TRIS, pH 8.0, 1 M NaCl, 5 mM β-mercaptoethanol, 1 mM MgCl_2_, 5 mM imidazole, 1 mM phenylmethane sulfonyl fluoride (PMSF) and a pinch of lysozyme. Cells were lysed by sonication at 4°C and the insoluble fraction was separated by centrifugation at 18,000 rpm (SS-34 Sorvall) for 30 minutes. The soluble fraction was passed over pre-equilibrated Ni-NTA resin (Qiagen), washed with 20 mM TRIS, pH 8.0, 1 M NaCl, 5 mM β-mercaptoethanol and 30 mM imidazole. Protein was batch eluted with 20 mM TRIS, pH 8.0, 1 M NaCl, 5 mM β-mercaptoethanol and 450 mM imidazole. Eluted protein was mixed with 500 µg of previously purified SUMO1 protease. The cleavage reaction was dialyzed overnight at 4°C into 20 mM TRIS, pH 8.0, 1 M NaCl, 5 mM β-mercaptoethanol. Purified EBNA1 was obtained by passing the cleavage reaction over pre-equilibrated Ni-NTA resin. For crystallography experiments, the protein was further purified using a size exclusion column (HiLoad 26/60 Superdex 75 pg; GE Life Sciences)) equilibrated with 1 mM Hepes, pH 7.2, 10 mM dithiothreitol, and 500 mM NaCl. After running fractions on a Bis-Tris acrylamide gel to verify size and purity, protein was pooled, concentrated, aliquoted and frozen at - 80°C for long-term storage.

### Crystallization, data collection and structure solution of EBNA NPC

Purified EBNA1 from NPC at 5.3 mg/mL was incubated with a 1.5x molar excess of the single EBNA1 dimer binding site 5’ -GGATAGCCT ATGCTACCC-3’. Crystals were grown in 48 hours at room temperature in 0.2 M ammonium acetate, 0.15 M magnesium acetate tetrahydrate, 0.05 M Hepes, pH 7.0 and 5% PEG 4000 (Natrix condition 38, Hampton Research). Crystals were frozen in oil. Data was collected from a single crystal kept frozen with a Oxford Cryosystems Cobra system at 100° K on a Rigaku MicroMax-007 HF rotating anode X-ray generator (wavelength 1.54178 Å) with VariMax optics and using a Saturn 944 HG CCD detector. Data was indexed, reduced, and scaled using HKL3000. The structure was solved by molecular replacement using PHASER integrated into PHENIX [52] with 1B3T [53] as a search model. Models were refined in PHENIX using simulated annealing, minimization and individual B-factor refinement. Between refinement cycles, the model was manually rebuilt using the program Coot [54]. Data collection and refinement statistics are summarized in Table S1.

### DNA binding experiments on biacore

5’ biotinylated DNA corresponding to a single EBNA1 dimer binding (5’-GGGAAGCATATGCTTCCC-3’, from 1B3T) and two EBNA1 dimer binding sites (5’-CGATAG CATATGCTTCCCGTTGGGTAACATATGCTATTG-3’, dyad symmetry 3+4) were purchased from Integrated DNA Technologies. DNA was coupled to Biacore T200 compatible SA sensor chip (GE Healthcare Life Sciences). Binding between EBNA1 (0 nM to 200 nM) and these DNAs was performed in 20 mM tris, pH 8.0, 250 mM NaCl, 5 mM β-mercaptoethanol, and 1 mM MgCl_2_. Resulting sensorgrams were used to obtain a K_D_ plot ( [EBN1-DNA] *versus* [EBNA1]) and fit for K_D_ (nM), R_max_, and Chi^2^ using the Biacore T200 Evaluation Software (GE Healthcare Life Sciences) (Supplementary Figure 1). Chi^2^ is less than 10% of R_max_ for an acceptable data set.

### Recombinant EBV genomes

Recombinant EBV B95-8 and M81 genomes carries the prokaryotic F factor origin of replication, the green fluorescent protein (GFP) gene, the chloramphenicol (CAM) resistance gene, and the hygromycin (HYG) resistance gene as described previously [55, 56]. The B95-8/npcEBNA1 genome was obtained by homologous recombination exchanging the DNA binding domain of the EBNA1 gene (M81 coordinates 97101 to 97551; GenBank accession number KF373730.1) using two-step red recombination method [57]. Positive selected clones were purified and analyzed with the BamHI restriction enzymes to confirm correct recombination, which was further confirmed by Southern blotting of Terminal and IR1 (W) repeats, and by Sanger sequencing of the recombined region.

### Virus producer cell lines

EBV producer cell lines and virus were generated as described previously [58]. Briefly, rB95-8, rM81 and B95-8/npcEBNA1 Bac DNA was purified from 4ml of bacterial culture according to Bacmid purification kit (Nucleo Bond 740579, Clonetech) and the DNA was transfected into HEK293 cells (1mg DNA per 4×10^5^ cells) using lipofectamine 2000 (Invitrogen). Cells were seeded onto 150 mm culture plates in RPMI supplemented with 10% FBS and hygromycin (100 mg/ml). Eight GFP-positive colonies were expanded four weeks post transfection and tested for their ability to produce virus upon induction of the lytic cycle (Western blot, qPCR, and PFGE analysis) and selected for viral production.

### Recombinant virus production

Recombinant EBV was produced as described previously [58]. Briefly, 293 producer cells for rM81, rB95-8 or B95-8/npcEBNA1 were induced by transfection of a BZLF1 expression plasmid with co-transfection of a BALF4 expression plasmid. The supernatants were collected 4 days post-transfection and filtered through a 0.45mm filter to remove the cell debris. To concentrate virus the supernatant was layered onto a 10% sucrose cushion and spin at 26,000 rpm for 1h and virus pellet was resuspended in RPMI medium. Viral gene equivalents were determined by qPCR and by infection of 10^5^ Raji B cells with concentrated virus stock using quantitation methods described [34].

### Primary B cell infections and colony formation assays

B cells were purified from PBMCs (Peripheral blood mononuclear cells) using B-cell enrichment Kit II (Cat# 19054; Stem Cell Technologies). 2 x10^6^ B-cells were infected with 10 infectious particle units of recombinant virus at room temperature. After 2 weeks post infection, B-cell colonies were identified and counted by light microscope using a colony counting macro written for NIH Image.

### *in situ* proximity ligation assay (*in situ* PLA)

Cells were harvested, washed in PBS, and mounted onto slides by cytospin (Shandon Cytospin 3; Thermo Fisher) at 1,000 rpm for 5 min, were immediately fixed in 4% PFA (4% formaldehyde in PBS) on ice for 30 min and thereafter subjected to *in situ* PLA using Duolink Detection kit (Olink Bioscience, Uppsala, Sweden) according to the manufacturer's instructions for Duolink Blocking solution and Detection protocol. Briefly, slides were blocked, incubated with antibodies directed against mouse-anti EBNA1 (clone E1-2.5; Acris GmbH) and rabbit-anti Survivin (Novus biologicals; NB500-201) and thereafter incubated with PLA probes, which are secondary antibodies (anti-mouse and anti-rabbit) conjugated with oligonucleotides PLA probe minus and PLA probe Plus). Circularization and ligation of the oligonucleotides was followed by an amplification step. Slides were mounted using Vectashield (Vector Laboratories Inc, Burlingame, CA). Images were captured with a 63X lens on a Leica SP5 II Confocal microscope (Leica Microsystems) using LAS AF software for image processing and quantification. The number of foci, visualized as bright fluorescent signals, was counted in 10-15 cells/slide. n (number of slide) = 3

Additional Methods are provided in Supplemental Materials.

## SUPPLEMENTARY MATERIALS FIGURES AND TABLES


