# Correction: Carcinoma-risk variant of EBNA1 deregulates Epstein-Barr Virus episomal latency

**DOI:** 10.18632/oncotarget.27073

**Published:** 2019-07-09

**Authors:** Jayaraju Dheekollu, Kimberly Malecka, Andreas Wiedmer, Henri-Jacques Delecluse, Alan K.S. Chiang, Dario C. Altieri, Troy E. Messick, Paul M. Lieberman

**Affiliations:** ^1^ The Wistar Institute, Philadelphia, PA, USA; ^2^ Deutsches Krebsforschungszentrum, Heidelberg, Germany; ^3^ Department of Pediatrics and Adolescent Medicine, The University of Hong Kong, Hong Kong

**This article has been corrected**: Due to errors in image selection, Figure 5A shows an inadvertent duplicate of Donor 2 for M81. Figure 7D shows the Actin control from Donor 2 that was inadvertently duplicated from the Donor 2 Actin control shown in Figure 4 (which is the same extract and donor sample). Figure 4 is correct and unchanged. The corrected Figure 5 and Figure 7 are shown below. The authors declare that these corrections do not change the results or conclusions of this paper.

Original article: Oncotarget. 2017; 8:7248–7264. 7248-7264 . https://doi.org/10.18632/oncotarget.14540

**Figure 5:**
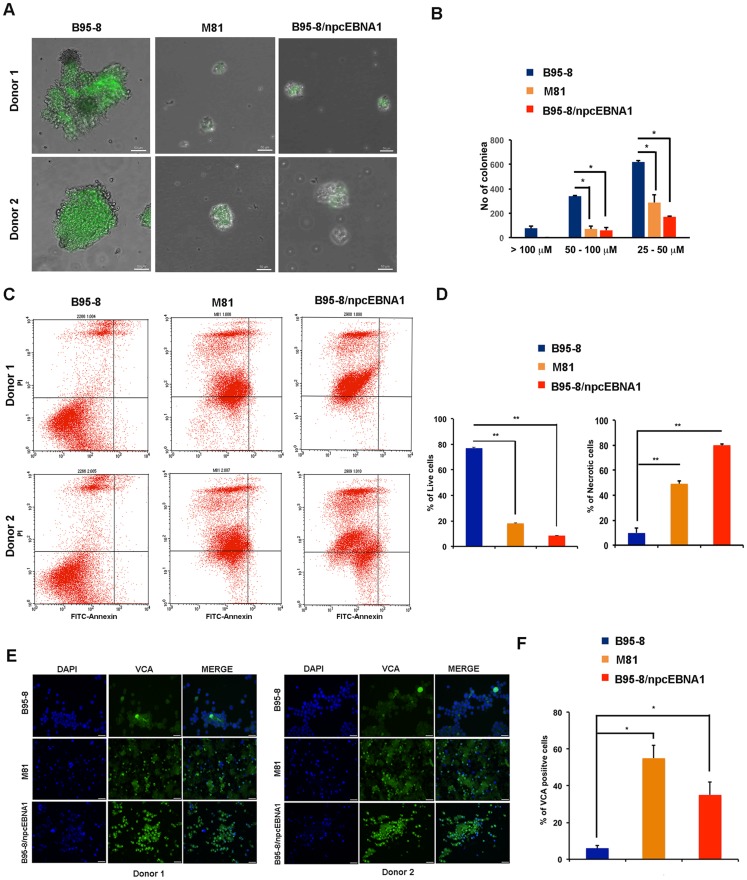
Defective B-cell blast formation by B95-8/npcEBNA1. Bacmid-derived virus for B95-8, M81, or B95-8/npcEBNA1 were assayed at 2 weeks post-infection of primary B-lymphocytes. **A.** GFP positive B-cell blasts for two independent donors were analyzed by high-throughput microscopy. **B.** The number of colonies imaged by microscopy with diameters of > 100, 50-100, or 25-100μM were quantified by Image J (panel B). **C.** B-cell blasts at 4 weeks post-infection were analyzed by FACS for apoptosis using propidium idodide (PI) (x-axis) and annexin V (y-axis). **D.** The percentage of live and necrotic cells assayed by FACS were quantified for two independent donors and three independent biological replicates.

**Figure 7:**
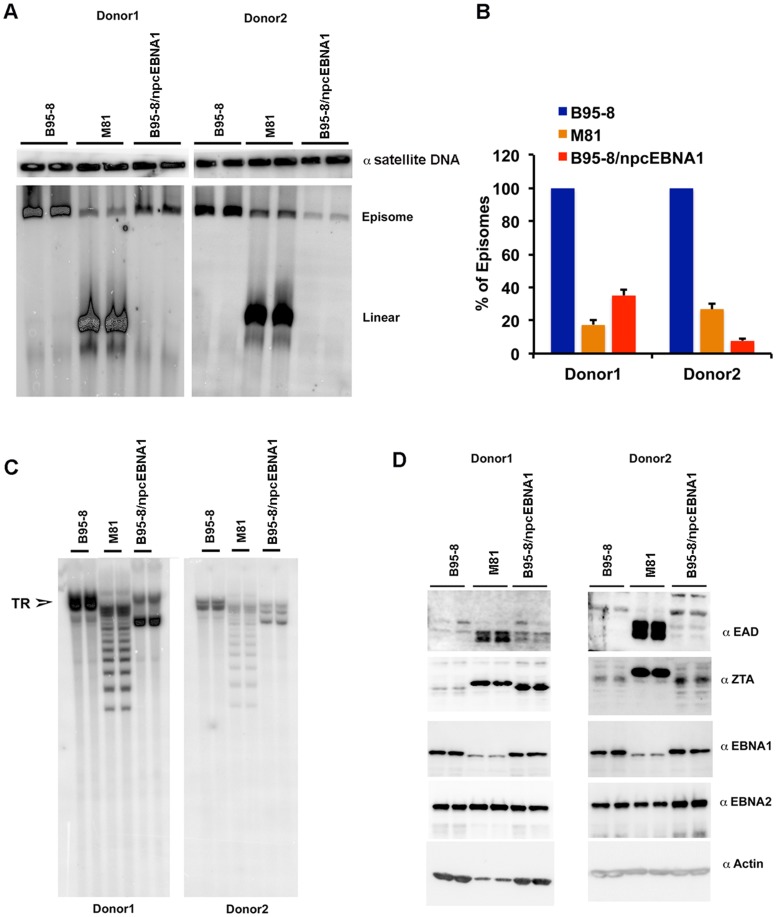
Low episome copy number and terminal repeat instability in LCLs with B95-8/npcEBNA1. **A.** PFGE analysis of LCLs generated with recombinant B95-8, M81, or B95-8/npcEBNA1 virus. Samples are run as technical replicates for two independent donor generated LCLs. Cellular α-satellite DNA is shown as loading control above each lane. **B.** Quantitation of EBV episomes relative to α-satellite DNA for PFGE shown in panel A. **C.** Southern blot analysis of EBV terminal repeats after digestion with BamHI for B95-8, M81, or B95-8/npcEBNA1 generated LCLs. **D.** Western blot for EAD, ZTA, EBNA1, EBNA2, and Actin for B95-8, M81, or B95-8/npcEBNA1 generated LCLs.

