# Supplementary material for: Carcinoma-risk variant of EBNA1 deregulates Epstein-Barr Virus episomal latency
Source: Oncotarget. 2017 Jan 6;8(5):7248–64. doi: 10.18632/oncotarget.14540 (PMC5352318; doi:10.18632/oncotarget.14540)
Supplement: Supplementary file 1 [file oncotarget-08-7248-s001.pdf]

Carcinoma-risk variant of EBNA1 deregulates Epstein-Barr Virus episomal latency

Jayaraju Dheekollu[1], Kimberly Malecka[1], Andreas Wiedmer[1], Henri-Jacques Delecluse[2], Alan K.S. Chiang[3], Dario C. Altieri[1], Troy E. Messick[1] and Paul M. Lieberman[1]

[1] The Wistar Institute, Philadelphia, PA USA

[2] Deutsches Krebsforschungszentrum, Heidelberg, Germany

[3] Department of Pediatrics and Adolescent Medicine, The University of Hong Kong, Hong Kong

Correspondence to: *Paul M. Lieberman,* ***email***: *Lieberman@wistar.org*

Keywords: epstein-barr virus (EBV), EBNA1, nasopharyngeal carcinoma (NPC), survivin, latency

Received: *October 29, 2016* ***Accepted***: *December 26, 2016* ***Published***: *January 06, 2017*

Supplemental Data

Crystallographic Information Table S1

Space Group	$P2_12_12_1$
Cell Dimensions	
a, b, c (Å)	60.0, 64.9, 111.7
α, β, ɣ (°)	90, 90, 90
Data Collection	
Resolution (Å)	50.0 - 2.30 (2.34 – 2.30)
Total Linear R factor	0.198 (0.879)
I/σ	21.9 (6.4)
Completeness (%)	98.7 (97.6)
Redundancy	7.2 (7.2)
Refinement	
Reflections	143442
R_{work}/R_{free}	0.1854 / 0.2329
Average B factor (Å^2)	22.3
Number of Atoms	
Protein	2272
DNA	732
Water	122
RMSDs	
Bond Lengths (Å)	0.91
Bond angles (°)	0.005

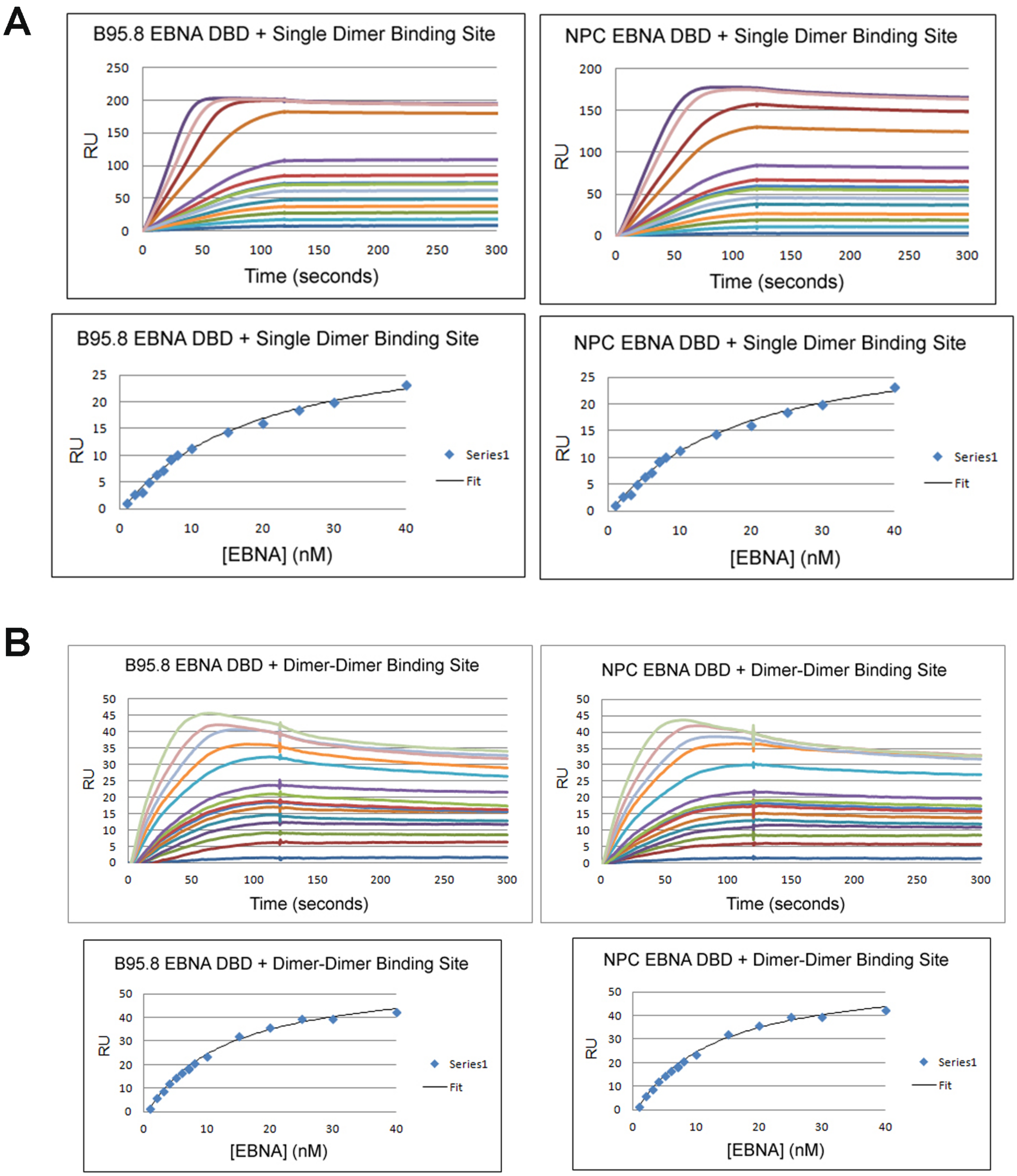

Figure S1: Surface Plasmon Resonance (SPR) analysis of EBNA1 DNA binding properties. Representative sensorgrams using for single-dimer (A) or dimer-dimer (B) binding sites the Biacore T200 Evaluation Software (GE Healthcare Life Sciences).

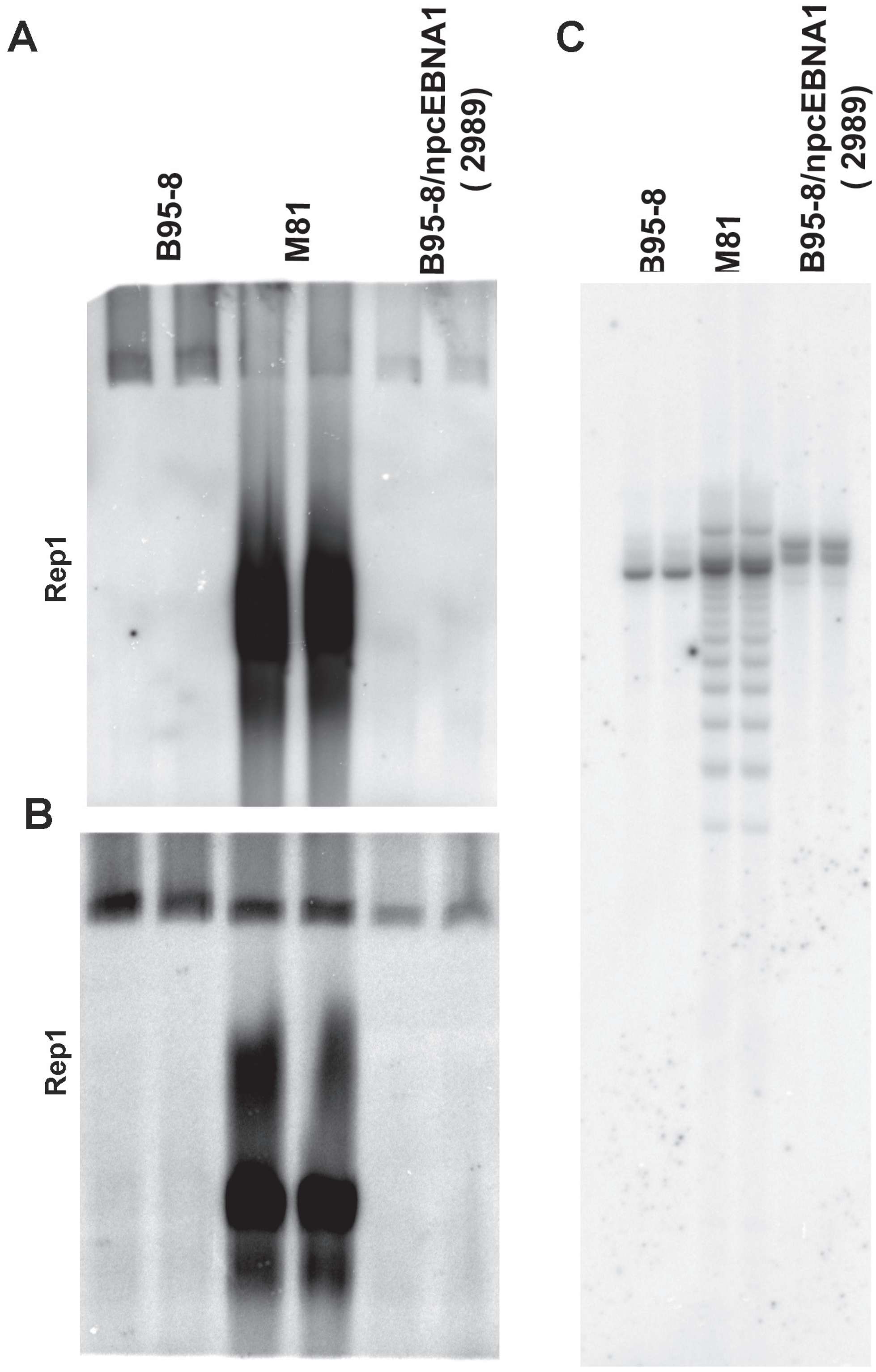

Figure S2: Characterization of a second recombinant B95-8/npcEBNA1 (2989) in episome maintenance and terminal repeat stability. (A) PFGE analysis of LCLs transformed with B95-8, M81, or B95-8/npcEBNA1 (2989) bacmid derived virus. (B) Biological replicate of experiment shown in panel A. (C) Southern blot of terminal repeats from LCLs shown in panel A.

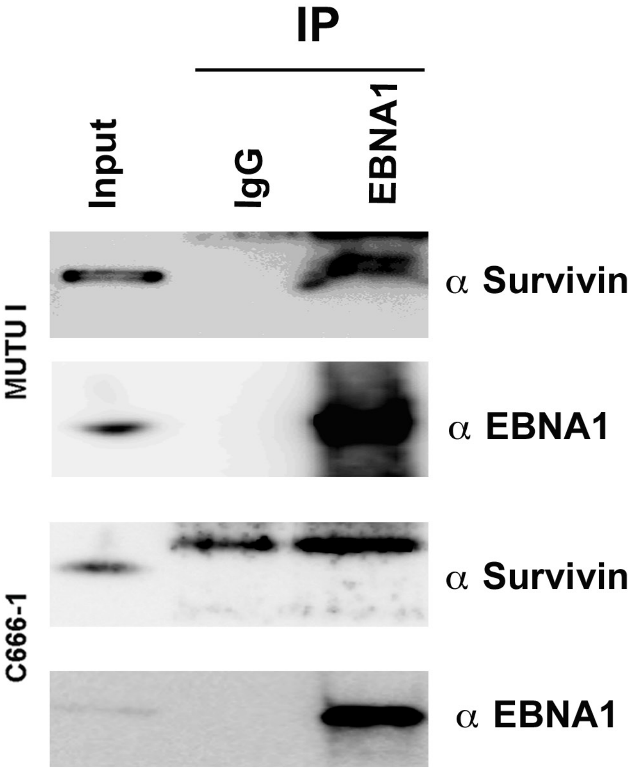

Figure S3: EBNA1 coIP interaction with Survivin. MUTU I cell (top panels) or C666-1 cell (lower panels) extracts were subject to IP with antibody to EBNA1 or control IgG, and then assayed by Western blot with antibody to Survivin or EBNA1, as indicated. The data is a biological replicate of that shown in Figure 8D, except that input levels of EBNA1 are not adjusted relative the input levels for Survivin (5% input for all samples).

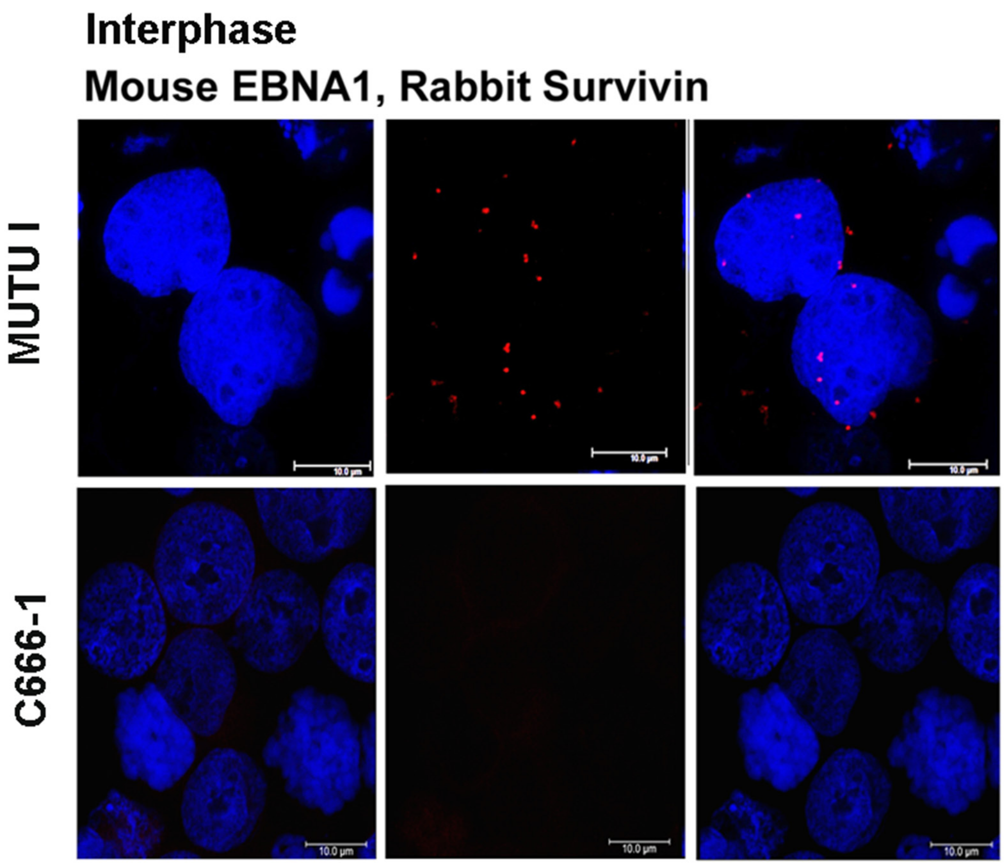

Figure S4: In situ Proximity Ligation Assay in Interphase cells for MUTU I or C666-1, as indicated (as described in Figure 7F and G).

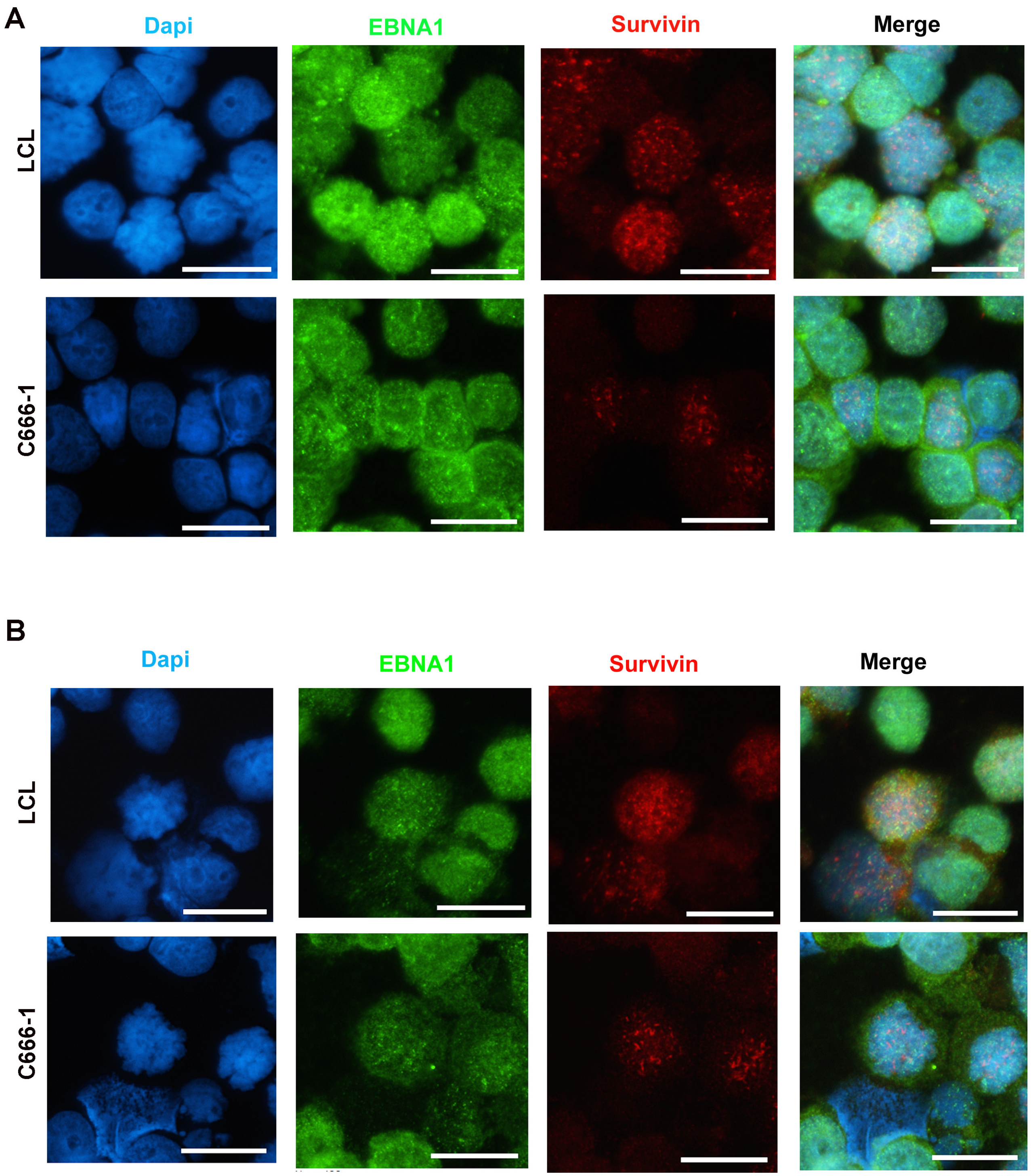

Figure S5: Control Immunofluorescence showing signal strengths for in situ Proximity Ligation Assay. Dapi (blue), EBNA1 (green), and Survivin (red) are shown in B95-8 LCL or C666-1 with indirect immunofluorescence using same primary antibodies as used in in situ PLA shown in Figure 8F. Scale bar= 50 µM.

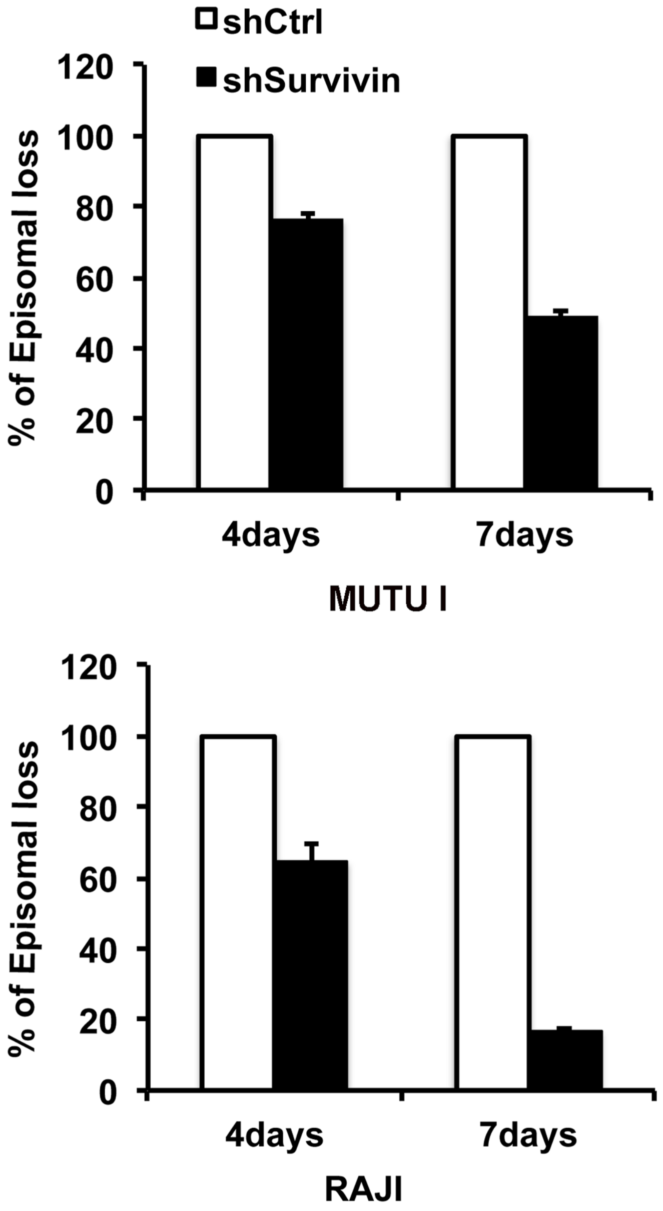

Figure S6: Quantification of EBV episomes from pulse field gel electrophoresis and Southern blot analysis. PFGE Southern blots shown as representative images in Figure 8H were quantified by PhosphorImager for biological replicates harvested at 4 days or 7 days post-transduction with lentivirus shCtrl or shSurvivin in either MUTU I (top) or RAJI (lower) cells.

Supplemental Methods

Plasmid DNA replication assay

Plasmid DNA replication assays have been described previously (1). Essentially, HeLa and SUNE1 cells were transfected with 10μg of OriP plasmids expressing either FLAG-B95-8 EBNA1 or Flag-NPC EBNA1. Cells were split after 24 h, and then harvested at 72 h post transfection cells. Episomal plasmid DNAs were extracted by Hirt Lysis method (2). Extracted DNAs were digested with either *Bam*HI or *Dpn*I, and *Bam*HI overnight at 37°C. Digestions were extracted with phenol:chloroform (1:1), precipitated and electrophoresed on a 0.8% agarose gel. Southern blot was performed as previously described (3) and Southern blots were visualized and quantified by PhosphorImager analysis using a Molecular Dynamics Storm PhosphorImager (Molecular Dynamics, Sunnyvale, CA).

Colony formation assays

HeLa and SUNE-1 cells were co-transfected with 10 μg of either Flag-EBNA1 (B95-8) or Flag-EBNA1 (NPC) contain OriP plasmids. The cells were split after 24 h and selected with 0.5 μg/ml hygromycin. After two weeks of selection, the resulting hygromycin-resistant colonies were stained with toluidine blue (0.5mg toluidine blue in 2% sodium carbonate). Colonies that were at least 2 mm in size were scored as positive. Colonies were counted using a colony counting macro written for NIH Image as described previously [1,2].

Plasmid maintenance

HeLa and SUNE-1 cells were transfected with 10µg of either OriP plasmids co-expressing Flag-B95-8 EBNA1 or Flag-NPC EBNA1. Cells were split after 24 h and were selected for 7 days with hygromycin. Cells were collected flow sorting of GFP expressing cells and equal amount of GFP positive cells were re-plated. After two weeks, plasmid DNA was extracted and digested with BamHI. Southern blot analysis was performed as above.

shRNA-mediated knockdown

EBV-positive cells were infected with lentivirus expressing shSurvivin, or shControl shRNA by spin infection. After 48 h postinfection and then selected with puromycin for 72 h.

RT-qPCR

Reverse transcription (RT)-qPCR assay of viral gene expression was performed as previously described (3).

Pulse Field Gel Electrophoresis (PFGE)

Cells were resuspended 1× phosphate-buffered saline (PBS) and an equal amount of 2% agarose to form agarose plugs containing 1×10^6 cells that were then incubated for 48 h at 50°C in lysis buffer (0.2 M EDTA [pH 8.0], 1% sodium sarcosyl, 1 mg/ml proteinase K). The agarose plugs were washed twice in TE buffer (10 mM Tris [pH 7.5] and 1 mM EDTA). Pulsed-field electrophoresis was performed as described previously for 23 h at 14°C with a linear ramping pulse of 60 to 120 s through 120°C (Bio-Rad CHEF Mapper) (3). DNA was transferred to nylon membranes by established methods for Southern

blotting (4). The DNA was then detected by hybridization with a ^{32}P-labeled probe specific for the EBV WP region and visualized with a Molecular Dynamics PhosphorImager.

Immunoprecipitation

Cells were extracted with lysis buffer (20 mM Tris-HCL, pH 7.4, 1 mM EDTA, 0.1 mM EGTA, 2 mM MgCl2, 150 mM NaCl, 1 mM Na3VO4, 1 mM NaF, 20 mM sodium glycerophosphate, 5% glycerol, 1% TritonX100, 0.5% Sodium dodecyl sulfate, 1× protease inhibitors (Simga), 1× Phosphatase inhibitors (Sigma) and I mM PMSF). After rotation for 60 min at 4°C, the lysate was centrifuged for 20 min at 16,000× g, and the supernatant was recovered. The cleared extracts were used for immunoprecipitation with antibodies as indicated.

Annexin V staining

Cell apoptosis was determined by the Annexin V Apoptosis Detection Kit APC (eBioscience Inc.) according to the manufacturer's protocol. Approximately 1×10^6 cells in each experimental group were washed with PBS and then resuspended in 100 ul of Annexin binding buffer (100 mM HEPES pH 7.4, 140 mM NaCl and 25 mM $CaCl_2$) containing Annexin V (5 µl) and PI (5 µl) were added to each sample. After 30 min, the cells were diluted with binding buffer and then analyzed by flow cytometry.

Supplementary References

1. **Deng Z, Lezina L, Chen CJ, Shtivelband S, So W, Lieberman PM.** 2002. Telomeric proteins regulate episomal maintenance of Epstein-Barr virus origin of plasmid replication. Mol Cell **9:**493-503.
2. **Hirt B.** 1967. Selective extraction of polyoma DNA from infected mouse cell cultures. J Mol Biol **26:**365-369.
3. **Dheekollu J, Lieberman PM.** 2011. The replisome pausing factor Timeless is required for episomal maintenance of latent Epstein-Barr virus. J Virol **85:**5853-5863.
